# Sanitary Reform in Relation to Social and Moral Development

**Published:** 1856-04

**Authors:** Benjamin Daniel


### SANITARY REFORM IN RELATION TO SOCIAL AND MORAL DEVELOPMENT.

By BENJAMIN DANIEL, M.R.C.S.

If the aphorism, that the proper study of mankind is man, be true in a general sense, how much more is it applicable to those whose proper duty it is to investigate the causes of his diseases, to trace them to their consequences, and more or less to obviate or modify their injurious influences. Such a study resolves itself into an acquaintance with the natural constitution of man, and with the nature of those circumstances by which he is encompassed; how far he is subject to them, and how far he has the capability of controlling them. Without such a comprehensive view, it would seem somewhat presumptuous to interfere with the delicate mechanism by which natural functions (whether in health or disease) are performed; and a careful consideration of the quality of the organ, and that of the remedial agent, with an accurate experience of their mutual relations, can alone form an established basis for the foundation of sanitary science. At a time like the present, when social questions occupy so much of men's minds, much assistance may be given in forwarding their solution, by a due appreciation of the connexion which exists between physical requirements and moral development. It is not enough to regard the subject of our study simply anatomically, as so many nerves, so many arteries, so many bones and muscles, arranged so and so; or chemically to look upon him as a laboratory for the composition or decomposition of elementary bodies; or as composed of so much sulphur, so much phosphorus, so much lime. Nor will it do to limit our observations to the structural changes which take place under the normal or morbid influence of organic life. It is necessary that we also take into consideration his constitution as a moral and intelligent being. In the earlier stages of society, when manners were more simple and diseases more rare, the profession of medicine was combined with that of the priest; and Celsus tells us that in his days health had degenerated, and a complicated system of medicine had become necessary, on account of the effects of sloth and luxury upon the Romans; and that all the means of art were unavailable to counteract those evil results. I believe it to be capable of demonstration, that neglect of the physical requirements of the body is followed or accompanied by moral delinquency; and I believe, also, that these two conditions react upon each other, and that, by their reciprocal and combined influences, human nature might become so degraded, as to justify the Mephistophelian observation, that "the only use mankind makes of his reason, is to make himself more exquisitely animal than any other living creature". It is a bounden duty to probe the nature of existing evils, in order that remedies should be found. It may be more convenient, and add more to our complacency, to ignore the existence of unpleasant subjects; but it is not always safe to do so. We are told that "the turning away of the simple shall slay them, and the prosperity of fools shall destroy them."

Whatever may be the moral and religious requirements of man, he has qualities, in common with all animal nature, which imperiously demand gratification. Hunger and thirst will be satisfied at all risks; and wolfish ferocity has been observed under their influence. It is a radical error to suppose that, as a general rule, one can neglect the body, and at the same time develope the intellect and elevate the soul. We are not endowed with passions, with inclinations, or antipathies, that they should go for nothing, or be artificially destroyed, and that we should become, like the Stoics, simply intellectual and mechanical; nor does religion teach us to neglect those things which are necessary for the body, whilst we pay exclusive attention to those which are requisite for the soul. A code of sanitary laws in relation to religion and to society exists in the Pentateuch; and it is a subject of careful reflection how far they were adapted to the regeneration of a people which had become demoralised and servile under the influence of oppression. According to profane writers, and the internal evidence of the sacred records, the Jews were subject to leprosy; and in Leviticus xiv, 35, etc., minute regulations are given for the prevention of the spread of that disease. When the leprosy had localised itself in any particular dwelling, it was to be eradicated by cleansing and whitewashing, which, if ineffectual, was to be followed by the destruction of the unhealthy dwelling house. Leprosy appears to have been a disease of a very malignant character, according to the stringent regulations which were enacted for its eradication. The symptoms are also described with minute accuracy, to enable the priest to form a correct diagnosis.

Tacitus, remarking on the subject of the Exodus observes, "Very many historians agree, that a hideous pestilence having broken out in Egypt, king Bocchoris, eager to obtain a remedy, consulted the oracle of Hammon, which commanded him to purify the land by expelling into other countries that people (the Israelites), which was hateful to the gods. They were therefore gathered from all parts, and being driven into a dreary desert, and breaking out into despair and lamentations, Moses alone of all the exiles exhorted them not to expect any assistance either from the gods or from men, as they had been deserted by both, but only to trust themselves to him as a celestial leader by whose aid they had already conquered their present miseries. They assented, and commenced in perfect ignorance their planless march." Cleanliness is impressed in almost every page of the Levitical code, constant washings and bathings are commanded after contact with any sort of uncleanness, — whether it consisted in contact with the sick, or with dead bodies, or with unclean beasts, or from any personal pollution of any description. Cleanliness was made an article of religion second only to godliness. Personal purity of the body amounted almost to sanctification, and was made emblematical of purity of soul. To avoid the accumulation of filth in the camp, it was ordered, "Thou shalt have a place also without the camp, whither thou shalt go forth abroad. And thou shalt have a paddle upon thy weapon; and it shall be when thou wilt ease thyself abroad thou shalt dig therewith, and shalt turn back and cover that which cometh from thee." Restrictions in the nature of the material of which their clothing was to be composed,* and restrictions in the nature of food, were institutions adapted to curb extravagance in dress and diet. In the latter particular it would seem that the chosen people, in their passage through the desert, were almost incorrigible. They continually rebelled, fearing hunger and thirst. Manna was sent to appease them. They even got tired of this heaven-sent food; it became insipid to them, and they craved for something more savoury; for we read that "They wept again and said, there is nothing at all but this manna before our eyes," and they longed for "the cucumbers and the melons, and the leeks and the onions and the garlic." To punish them quails were sent, on which they feasted, and in consequence suffered from the visitation of a plague.

The nature of an eastern climate renders a particular attention to the requirements of the body imperative, the action of the skin being so much more vigorous in those regions. Disorders of the stomach, liver, and bowels are also frequent, and are liable to be caused by improper or excessive diet. It is hence common to most orientals to observe religious restrictions in their food. The Brahmins adopt the vegetarian system, and Goldsmith addresses them with, "Hail, O ye simple honest Brahmins of the East, ye inoffensive friends of all that were born to happiness as well as you. You never sought a short-lived pleasure from the miseries of other creatures, you never studied the tormenting arts of ingenious refinement, you never surfeited upon a guilty meal." It is said that the ancient Syrians and Egyptians did not eat fish, and that the ancient Greeks also avoided it from superstitious motives; that the Egyptians of Thebes

---

* Deut. xxii, 11.

did not eat sheep out of respect to their god "Hammon," whom they worshipped in the shape of a ram. It is remarkable that many of the traditionary customs of the modern Jews, are recorded by Herodotus as the particular manners of the Egyptians: thus he says, "that no Egyptian man or woman will kiss a Grecian on the mouth, or use the knife, spit, or cauldron of a Greek, or touch of the flesh of a pure ox that has been divided by a Grecian knife." "That the Egyptians were circumcised for the sake of cleanliness, thinking it better to be clean than handsome." (Euterpe ii, § 47.) They considered the pig an abomination; and if by accident an Egyptian should touch one only with his garment, "he forthwith goes to the river and plunges in." In regard to their habits of cleanliness, and their notions of the causes of disease and the means of preventing it, he observes, "that they dressed themselves in linen garments, which were constantly well washed," that "they purged themselves every month three days successively, seeking to preserve health by emetics and clysters; for they supposed that all diseases to which men are subject proceed from the food they use." The priests went so far as to shave their whole body every third day, that neither lice nor any impurity might be found upon them. When engaged upon the service of the gods, they washed themselves in cold water twice every day and twice every night. Some colour is lent to the necessity of these extreme precautions by the letters we receive from the East. The stench of a Turkish camp is recorded as something appalling; whilst, in the camp before Sebastopol, the animated nature to be seen on a soldier's under-garment has been said to lie as thick as the small print of a newspaper.

How far sumptuary laws are capable of moderating luxury in diet or in costume is very doubtful. To eat turbot and to dress in silk was at one time the prerogative of princes, whilst now it is the prerogative of those who can afford it; and we find that, as a general rule, the very prohibition acts as a stimulant to the appetite, and gives a relish to that which otherwise would be uncared for. Whatever practical examples the great ones of the earth afford, are quickly taken up by the people who follow in their wake. *Quidquid principes faciunt, præcipere videntur.*

In the matter of diet, the ages that can be said to equal our own in the ingenuity of their *cuisine*, and the variety of *mets* and *entremets*, appear to be, that which called forth the laws of Lycurgus for the reform of the Spartans, and that which obtained in the time of Nero, in Rome. According to all historic accounts at such periods, men seem to have justified Shakespeare's conclusion :—

> "What is a man
> If his chief good and market of his time
> Be but to sleep and feed? A beast, no more."

At the time that Lycurgus made his laws, Plutarch tells us that even then, 800 or 900 A.C., social evils were rampant, luxury and pride possessed the rich, misery and want, with its concomitant debasement, was the lot of the poor; therefore, to prevent hoarding of wealth, he banished the precious metals, and made iron the current coin of the realm. To check gluttony he established public eating-houses, where men met to a wholesome repast, composed of the monthly contributions of each member, which consisted of a bushel of wheat, eight gallons of wine, five pounds of cheese, two pounds and a half of figs for dessert, and a little money to buy fish and flesh; thus, in substantial nutriment, the Spartan fare was not wanting. A good appetite was considered the best sauce, and as all eating in private was strictly prohibited, the guests arrived at table tolerably hungry. A certain king of Pontus, having heard "black broth" much spoken of, sent for a Lacedemonian cook on purpose that he might have the privilege of tasting the genuine production, which, however, not coming up to his expectations, he hesitated not to discover his dislike; which induced the uncourteous cook to reply, that to make the broth relish, his Majesty would have required to bathe himself first in the river Eurotas. Plutarch states that "gormandising previously prevailed to such an extent, that the butchers and cooks used to cram the people in private, as they did the beasts and poultry they fed on. By this way of life their manners had become not only corrupted, but their bodies too were enfeebled, so that giving the rein to their sensual appetites, they stood in need of long sleep and hot baths; and, in a word, of as much care as if they were continually sick." Descriptions of a parallel state of society are to be found in Seneca's *Epistles*, and in Petronius Arbiter. The first expresses his opinions thus (epistle 21st): "Simple meats are out of fashion, and all are collected into one; so that the cook does the office of the stomach, nay, and of the teeth too, for the meat looks as if it were chewed beforehand": that "from these compounded dishes, arise compounded diseases, which require compounded medicines." He draws his inferences thus: "From hence come paleness, nervousness, and worse effects from indigestion than from a famine, weakness of the joints, the abdomen enlarged, suffusion of bile, torpor of nerves, and palpitation of the heart, with other diseases that are but the punishment of luxury." It was a reproach to Seneca, that he did not practise what he preached; but that is a failing very common amongst many who know what they ought to do, but do not choose to do it. "To say and do not", has been a practice from the earliest times; still, there was more sincerity about Seneca, than is to be found in Scribes and Pharisees in general. He was a man of the world, and he makes his confession (*Of a Happy Life,* cap. xv): "If I do not live as I preach, take notice, that I do not speak of myself, but of virtue; nor am I so much offended with other men's vices as I am with my own." Many moral and religious teachers of the present day are not half so candid. Dr. Johnson speaks of the courage of the adventurous experimentalist who first ate an oyster; and had he known how *patés de foie gras* were produced, he would not less have admired the science of modern cookery. A gross sensualism prevailed in Nero's day, and displayed itself in magnificent and *recherché* dinners. In a bill of fare may be observed these delicacies: a dish of grilled snails served up on a silver gridiron; dormice and blackbirds baked in a pie; hogs roasted whole, and filled with sausages and stuffed meats; the hinder paps of a sow which had only farrowed the day before; iced wines; and muscadine of a hundred years old.

In the present day, it is of some importance to trace what connexion there may exist between the social physical condition, and that restless longing for change or excitement which at the present time is more or less observed throughout the world. Nothing is more certain than that neglect of the essential conditions of health commonly reacts upon the moral system, begetting impatience, irritability, and want of self-control. An indigestion will make a wonderful difference in the look of the external world. An attack of gout is generally preceded, and frequently accompanied, by a most cynical spirit and irascibility. Jaundice is usually connected with the greatest despondency. A bilious look is commonly regarded as the feature of melancholy or discontent. Good assimilative powers make men contented and good humoured, friends of order and respectability. Thus Cæsar asks:—

> "Let me have men about me that are fat,
> Sleek-headed men and such as sleep o' nights;
> Yond' Cassius has a lean and hungry look:
> He thinks too much."

It is certainly true that physical discomfort begets an unquiet, peevish spirit. Children, suffering from chronic disorders of the bowels, become worn and haggard, and are most fretful, wayward, and uncontrollable. The reason why many people dread *ennui* as much as real pain, is that they are subject to an irritable, impatient, and restless spirit, combined with a body wanting physical stamina—a state of things which can only be remedied by a simpler manner of life. The popular remedy in such cases makes the case worse, as it is generally sought to be obviated by stronger and stronger doses of excitement. Under the influence of a temporary fit of the bile, a man will brood over his real or imaginary wrongs, until the phantasm assumes a reality too powerful for reason to withstand. It will assist the sufferer, to reflect that a physical cause is at the bottom of this moral disturbance:—

> "The yellow bile that in your bosom floats,
> Engenders all these melancholy thoughts."

And if an uncomfortable sensation cannot be reasoned away, it is encouraging to know that it is only of a temporary character. The stern, unbending constitution of a man's temper, may be temporarily aggravated by constipation of the bowels; and though it may appear a paradox, yet instances of the administration of justice, under such circumstances, have assumed the character of a wrong. Bichat, with some reason, has endeavoured to locate the seat of the emotions in the thoracic and abdominal viscera, and it is not without a foundation on the common practice of mankind; for the hand is carried to the head to indicate thought, but the expression of feeling and truth is ever shown by putting it upon the breast. In scriptural phraseology, to express the emotion of affection, it is said of Joseph that "his bowels did yearn upon his brother."

There is no question of the fact, that the occupations of modern civilised life are not such as to render the greatest amount of moral or physical well being. Sedentary occupations, by precluding the possibility of fresh air and exercise, generate a languid circulation, and a torpor of the general system; and during the hours of relaxation, which are generally in the evening, recreation is more frequently sought in the theatre, the concert room, and in the tavern, than in the quiet and comfortable contemplation which a well balanced constitution is able to draw from more intellectual pursuits. Every medical man has had occasion to remark how the soul may be bowed down under temporary bodily disorder, and to observe mental depressions which no intellectual strength can dominate; to hear sensations described, which hang like a millstone round the neck, making life itself a burthen which, but for religious principles, might gladly be laid down. Is it, then, to be wondered at, that to escape from such moral depression, some exciting distraction is eagerly sought after? Pleasure is less often sought for itself, than as a means of killing time.

Dr. Waller Lewis, having been commissioned by the Government, has sent in a report of the laws and ordonnances in France for the regulation of noxious trades and occupations. He speaks thus of the influences operating in manufacturing towns: "The population of the manufacturing towns are weak and diminutive; bent over the looms, and living in shade, they become etiolated like plants. Since the great increase of the manufactures in the department of the Haut Rhin (from 1810 to 1823), the average height of the people has not increased in the same proportion as in the neighbouring departments. Official documents prove that the population of the manufacturing towns is less vigorous than that of the rural districts. Everything tends to exhaust it: placed as auxiliaries by the side of the devouring activity of steam, or a fall of water which never reposes, it carries to its utmost limits the development of its forces; in the large assemblage of all ages and sexes, the passions are lighted up, the contagion of vice acts with a kind of furor, and the excesses of debauchery accelerate the deterioration of the strongest constitutions. In this manner the sources of reproduction become impoverished and corrupt; conceived in misery and libertinage, the weak scions of this bastardised population pass in their turns under the empire of the same causes of physical and moral degradation; it is, in fact, a circle without end, in which health and life go on exhausting themselves continually."

After such a statement, can it be doubted that the justice of inflicting punishment should be even questioned, when vice is fostered by the very institutions which develope material prosperity? No wonder that a knowledge of such influences acting upon the social system should make men cast about for a remedy. The establishment of reformatory schools has suggested itself, and, if properly carried out, may do some good. When it is stated that the moral bears a direct relation to the physical state (that is, taken as a general rule), it is not to be looked upon as an hypothetical supposition, but as a matter of the commonest observation, and is founded on the authority of reports to the Government, made by commissioners who state that, after thorough investigations, the result is invariable, that when food is dear, or when work is scarce, the moral thermometer falls, and committals for crime increase. Disease also shows itself in the shape of fever and dysentery; and the greater the privations, the greater the mortality. On the other hand, when necessaries are cheaper, and occupation brisker, an immediate fall of the number of committals and diseases is the direct result. It requires more than a vulgar philosophy to enable those who happen not to possess the necessaries of life, to maintain, under such unfavourable circumstances, an equable and virtuous mind. It is very well known, that the cause of the late French Revolution was not a simple question of a change of dynasty or of form of government, so much as it was an outburst of general discontent. St. James asks—"From whence come wars and rumours of wars among you? Come they not hence, even of your lusts that war in your members?" If, therefore, it can be shown that the influence of physical causes on the moral constitution is excited for good or for evil, and that power of selection is granted to us, it follows that the condition of society is dependent upon the wisdom which takes into consideration the full significance and importance of our natural requirements, and avails itself of so important an auxiliary of moral improvement. The laws of our physical nature cannot be abused or neglected with impunity; sooner or later they vindicate themselves, and the debt must be paid. It is advisable not to run up too long a score, or the reckoning may be more than we calculate.

---

